# A novel *DICER1* mutation identified in a female with ovarian Sertoli-Leydig cell tumor and multinodular goiter: a case report

**DOI:** 10.1186/1752-1947-8-112

**Published:** 2014-04-03

**Authors:** Maria Rossing, Anne-Marie Gerdes, Anders Juul, Catherine Rechnitzer, Martin Rudnicki, Finn C Nielsen, Thomas vO Hansen

**Affiliations:** 1 Center for Genomic Medicine, Rigshospitalet, Copenhagen University Hospital, Copenhagen, Denmark; 2 Department of Clinical Genetics, Rigshospitalet, Copenhagen University Hospital, Copenhagen, Denmark; 3 Department of Growth and Reproduction, Rigshospitalet, Copenhagen University Hospital, Copenhagen, Denmark; 4 Department of Pediatrics, Rigshospitalet, Copenhagen University Hospital, Copenhagen, Denmark; 5 Department of Obstetrics and Gynecology, Roskilde University Hospital, Roskilde, Denmark

**Keywords:** *DICER1*, Germ-line mutation, Multinodular goiter, Ovarian Sertoli-Leydig cell tumor

## Abstract

**Introduction:**

Germ-line mutations in the micro-ribonucleic acid processing gene *DICER1* have been shown to predispose to a subset of benign tumors susceptible to malignant transformation, including ovarian Sertoli-Leydig cell tumor, nontoxic multinodular goiter, multilocular cystic nephroma and pleuropulmonary blastoma, which can occur in children and young adults. This may be due to reduced Dcr-1 homolog expression in carriers of germline mutations, which causes impairment of micro-ribonucleic acid processing and deregulates the growth and differentiation of target cells, leading to an increased risk of tumorigenesis. Many carriers of germ-line *DICER1* mutations remain unaffected, but development of tumors within carriers is associated with varying prognoses.

**Case presentation:**

Despite the Dcr-1 homolog syndrome phenotype being incompletely defined, a *DICER1* mutation was suspected when a girl (case 1 patient) of Danish ethnicity presented with both an ovarian Sertoli-Leydig cell tumor and a multinodular goiter at the age of 13 years. In addition, family history included a male sibling (case 2 patient) who also had a multinodular goiter and had undergone a hemithyroidectomy at the age of 14 years. Subsequent *DICER1* screening of the girl identified two novel mutations in exon 21 - a nonsense (c.3647C>A, p.Ser1216*) and a missense (c.3649T>A, p.Tyr1217Asn) mutation. The siblings had inherited the mutations from their father and paternal grandfather, which both currently were asymptomatic, indicating reduced penetrance of the nonsense mutation. Analysis of the parents revealed that the mutations were present in *cis*, making the contribution of the missense mutation less significant.

**Conclusion:**

We report a novel pathogenic *DICER1* mutation (p.Ser1216*) in a Danish family associated with ovarian Sertoli-Leydig cell tumor and a multinodular goiter. A multinodular goiter was diagnosed in the siblings during childhood. Clinicians should be aware of a potential germ-line *DICER1* mutation when evaluating multinodular goiter in young patients with or without a family history of thyroid diseases.

## Introduction

Dcr-1 homolog (DICER1) is a member of the ribonuclease type III family and plays an important role in the processing and maturation of micro-ribonucleic acids (miRNAs) [1]. miRNAs are small (20 to 25 nucleotides), double-stranded, non-coding, endogenous RNA molecules that modulate gene expression at the post-transcriptional level by imperfect base pairing to the complementary sequences on target messenger RNAs (mRNAs). miRNA genes are transcribed by RNA polymerase II or III into primary miRNA transcripts termed pri-miRNAs. Pri-miRNAs are subsequently cleaved by the Drosha-DGCR8 complex to release hairpin-shaped pre-miRNAs. Pre-miRNAs are exported to the cytoplasm where DICER1 cuts their terminal loop and generates short miRNA duplexes. A single strand of the small RNA duplexes is finally incorporated into the RNA-induced silencing complex, and in this position, miRNAs bind to their target mRNAs, modulating protein expression [1].

Impaired DICER1 expression and subsequent altered miRNA processing have a substantial impact on the dysregulation of target oncogenes, leading to enhanced tumorigenesis [2]. *DICER1* mutations have previously been associated with ovarian Sertoli-Leydig cell tumor (SLCT), nontoxic multinodular goiter (MNG) and multilocular cystic nephroma. These conditions generally follow a benign course [3,4]. In addition, *DICER1* mutations predispose to a rare type of lung cancer most often seen in children, known as pleuropulmonary blastoma [5]. Recently, *DICER1* mutations were also suggested to be associated with diseases, such as Wilms’ tumor, cervix embryonal rhabdomyosarcoma and pineoblastoma [6-8].

Here we report a novel germ-line *DICER1* nonsense mutation in a pair of siblings with MNG, as well as SLCT in the index case.

## Case presentation

Case 1 patient was a 13-year-old girl of Danish ethnicity (proband), who presented with swelling of the neck, as well as a deep voice, hirsutism and acne vulgaris in the beard area of the face. She was subsequently diagnosed with MNG and ultrasonic examination identified 13 nodules ranging from 6 to 12mm in size. Examination of her hormonal status revealed increased levels of androstenedione (26nmol/l) and testosterone (total: 6.8nmol/l and free: 0.146nmol/l). Follicle-stimulating hormone and luteinizing hormone levels were normal, as was the Synacthen test. A computed tomography scan identified a tumor in her left ovary. She immediately underwent unilateral oophorectomy and subsequent histopathological examination detected encapsulated tumor tissue, including strings of immature and slightly atypical Sertoli cells together with accumulations of Leydig cells. There were only a few mitoses and no necrosis. The final histopathological diagnosis was reported as an encapsulated SLCT of intermediate degree of differentiation. Immunohistochemical analyses showed positive staining for vimentin and inhibin, whereas α-fetoprotein gave a negative result. Follow-up included an ultrasonic-guided examination of the ovary and measurement of hormonal status and serum inhibin B level for five years with an increasing interval.

**Figure 1:**
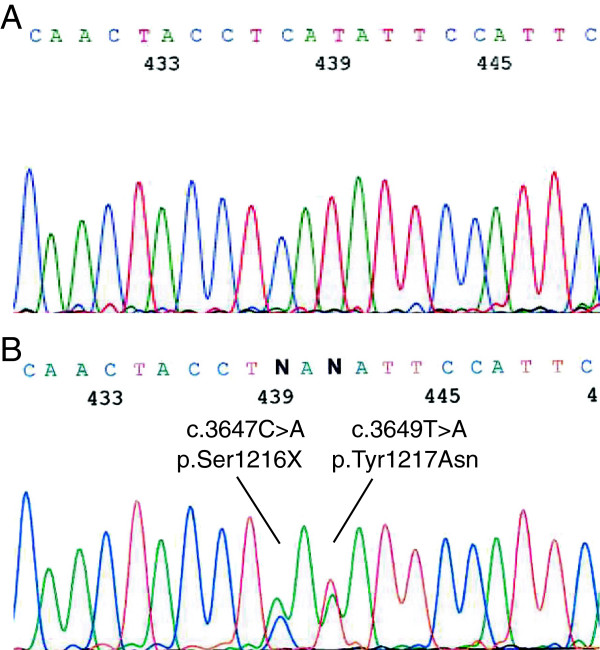
Identification of the*DICER1*c.3647C>A, p.Ser1216* and the c.3649T>A, p.Tyr1217Asn mutations. Deoxyribonucleic acid was purified from blood samples from a wild-type and the proband. The *DICER1* gene was amplified using intronic primer pairs flanking each exon, followed by sequencing. The analysis revealed a *DICER1* nucleotide c.3647C>A, p.Ser1216* mutation in exon 21 and a *DICER1* nucleotide c.3649T>A, p.Tyr1217Asn mutation also in exon 21 in the proband (panel **B**) not present in the wild-type (panel **A**).

Since our proband, had an ovarian SLCT as well as MNG, the pediatricians suspected a *DICER1* mutation and referred the girl for genetic counseling. Blood samples were collected, genomic deoxyribonucleic acid (DNA) was purified, and the entire coding region and the exon-intron boundaries of *DICER1* were screened. The analysis identified two mutations in exon 21 - a nonsense mutation (c.3647C>A, p.Ser1216*) and a missense mutation (c.3649T>A, p.Tyr1217Asn) of unknown significance (Figure 1, panel B).

**Figure 2:**
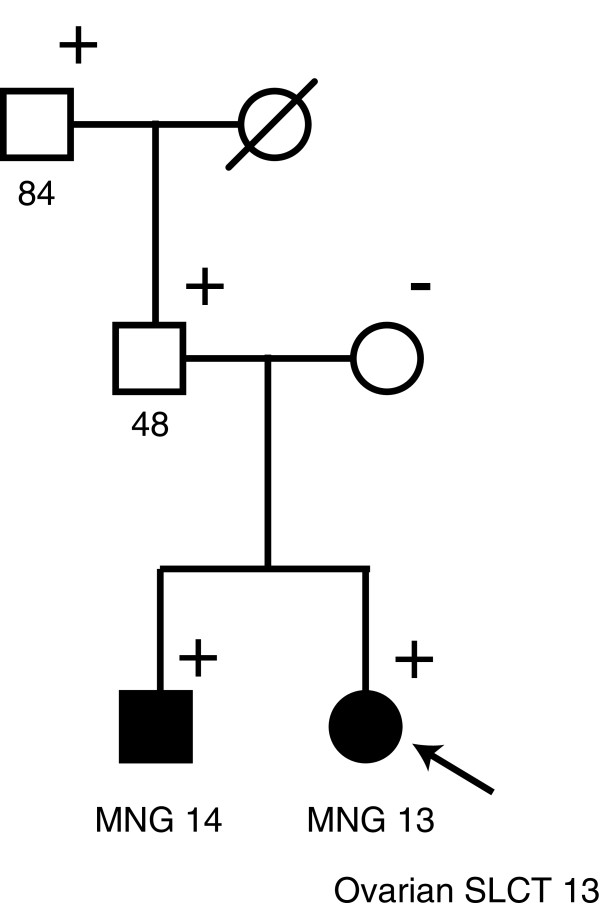
**Family pedigree.** Multinodular goiter and ovarian Sertoli-Leydig cell tumor are indicated as well as the age at diagnosis. Diagonal slash indicates deceased, while the proband is indicated with an arrow. Mutation-positive individuals are indicated with +. MNG, multinodular goiter; SLCT, Sertoli-Leydig cell tumor.

Since a pathogenic *DICER1* mutation was identified, first and second degree relatives of the proband were screened for the mutation. The analyses revealed that the brother, the father and the paternal grandfather all carried the pathogenic *DICER1* mutation as well as the missense mutation (Figure 2), indicating that the two mutations are present in *cis* on the same allele.

Case 2 patient is a five-year older brother of the proband who had an MNG and underwent hemithyroidectomy at the age of 14. Ultrasound (US)-guided fine-needle aspirate from the dominating hypoechoic cold nodule measuring (4×3.5×2.5cm) yielded follicular neoplasia. Subsequently, the brother underwent a right-sided hemi-thyroidectomy and the histopathological examination revealed an encapsulated follicular adenoma with papillary hyperplasia. The brother is at present time in his early 20s and remains euthyroid without any pressure symptoms. He has annual consultations regarding the MNG of the left lobe to monitor potential growth in the cold nodules.

The father, at the age of 50, has no chronic illnesses and no medical history of any thyroid symptoms. A recent thyroid check-up confirmed the euthyroid status and a normal palpatory examination of the thyroid and neck area. The medical history of the paternal grandfather reveals a remarkably fit patient in his mid-80s who is being treated for hypertension and hypercholesterolemia. The grandfather remains euthyroid and palpatoric examination of the thyroid and neck area did not reveal any goiter, nodules or enlarged lymph nodes.

## Discussion

Ovarian SLCTs are rare androgen-producing sex cord-gonadal stromal tumors that account for less than 1% of ovarian tumors, occurring most commonly in younger women while MNG is a common disease likely caused by low iodine intake but also with a heritable predisposition. In this study, a girl was diagnosed with MNG and ovarian SLCT at the age of 13 years. Subsequently, a pathogenic *DICER1* mutation was identified. To date, 45 different pathogenic germ-line *DICER1* mutations have been reported in 53 probands with various neoplasms worldwide, including frameshift, nonsense, splicing and missense mutations scattered throughout the gene, as well as large genomic rearrangements (Table 1) [3-5,7-13]. The novel nonsense mutation identified in the present study resides in exon 21, the largest exon of *DICER1,* where currently 11 other pathogenic germ-line mutations and deletions have been identified. Hence, exon 21 of the *DICER1* gene has the highest frequency of disease-causing mutations, followed by exons 8 and 23 with five pathogenic mutations each. Exon 21 is of particular interest as it encodes a large part of the ribonuclease (RNase) IIIa domain. The RNase IIIa domain is highly important for the production of miRNAs from the 3’ arm of precursors and forms, together with RNase IIIb, the core catalytic element of Dicer1.

**Table 1:** **Previously reported germ-line mutations in the** ***DICER1*** **gene**

**Region**	**Nucleotide change**	**Amino acid change**	**Reference**
Exon 4	c.328_338dupGTGTCAGCTGT	p.Arg114Cysfs*18	Slade *et al.*[11]
Exon 7	c.876_879delAAAG	p.Arg293Ilefs*4	Rio Frio *et al.*[4]
Exon 8	c.912_919dupAGACTGTC	p.Arg307Glnfs*8	Foulkes *et al.*[7]
Exon 8	c.1128_1132delAGTAA	p.Lys376Asnfs*11	Sabbaghian *et al.*[8]
Exon 8	c.1153delC	p.Arg385Alafs*73	Slade *et al.*[11]
Exon 8	c.1196_1197dupAG	p.Trp400Serfs*59	Slade *et al.*[11]
Exon 8	c.1306dupT	p.Ser436Phefs*41	Foulkes *et al.*[7]
Exon 9	c.1507G>T	p.Glu503*	Hill *et al.*[5]
Exon 10	c.1525C>T	p.Arg509*	Darrat *et al.* 2013
Exon 10	c.1684_1685delAT	p.Met562Valfs*11	Hill *et al.*[5]
Exon 10	c.1716delT	p.Phe572Leufs*15	Slade *et al.*[11]
Exon 12	c.1910dupA	p.Tyr637*	Doros *et al.*[10]; Hill *et al.*[5]
Exon 12	c.1966C>T	p.Arg656*	Foulkes *et al.*[7]; Slade *et al.*[11]; Hill *et al.*[5] (reported in four individuals)
Intron 12	c.2040+1G>C	p.?	Slade *et al.*[11]
Intron 13	c.2117-1G>A	p.?	Foulkes *et al.*[7]
Exon 14	c.2245_2248dupTACC	p.Pro750Leufs*12	Hill *et al.*[5]
Exon 14	c.2247C>A	p.Tyr749*	Doros *et al.*[10]; Hill *et al.*[5]
Exon 15	c.2268_2271delTTTG	p.Cys756*	Slade *et al.*[11]
Exon 15	c.2392dupA	p.Thr798Asnfs*33	Hill *et al.*[5]
Exon 16	c.2457C>G	p.Ile813_Tyr819del	Rio Frio *et al.*[4]
	r.2437_2457del21		
Exon 16	c.2516C>T	p.Ser839Phe	Rio Frio *et al.*[4]
Intron 17	c.2805-1G>T	p.Tyr936_Arg996del	Rio Frio *et al.*[4]
	r.2805_2987del183		
Exon 18	c.2830C>T	p.Arg944*	Hill *et al.*[5]
Intron 18	c.2988-2_2988-1delAGinsCT	p.?	Slade *et al.*[11]
Exon 21	c.3270-6_4051 – 1280delinsG	p.Tyr1091Ser*28	Sabbaghian *et al.*[13]
Exon 21	c.3288_3289insTTTC	p.Gly1097Phefs*8	Slade *et al.*[11]
Exon 21	c.3505delT	p.Ser1169Glnfs*23	Slade *et al.*[11]
Exon 21	c.3540C>A	p.Tyr1180*	Hill *et al.*[5]
Exon 21	c.3583_3584delGA	p.Asp1195Leufs*39	Slade *et al.*[11]
Exon 21	c.3611_3616delACTACAinsT	p.Tyr1204Leufs*29	Foulkes *et al.*[7]
Exon 21	c.3647C>A	p.Ser1216*	This study
Exon 21	c.3665delT	p.Leu1222Tyrfs*17	Slade *et al.*[11]
Exon 21	c.3726C>A	p.Tyr1242*	Slade *et al.*[11]
Exon 21	c.3793delA	p.Thr1265Glnfs*37	Slade *et al.*[11]
Exon 21	c.3907_3908delCT	p.Leu1303Valfs*4	Foulkes *et al.*[7]
Intron 21	c.4050+1delG	p.?	Foulkes *et al.*[7]
Exon 23	c.4309_4312delGACT	p.Asp1437Metfs*16	Bahubeshi *et al.*[3]; Doros *et al.*[10]
Exon 23	c.4403_4406delCTCT	p.Ser1468Phefs*21	Slade *et al.*[11]
Exon 23	c.4740G>T	p.Gln1580His	Slade *et al.*[11]
Exon 23	c.4748T>G	p.Leu1583Arg	Hill *et al.*[5]
Exon 23	c.5018_5021delTCAA	p.Ile1673Thrfs*31	Rio Frio *et al.*[4]
Exon 24	c.5104C>T	p.Gln1702*	Doros *et al.*[10]; Dehner *et al.*[9]
Exon 24	c.5122_5128delGGAGATG	p.Gly1708Argfs*7	Slade *et al.*[11]
Exon 25	c.5465A>T	p.Asp1822Val	Slade *et al.*[11]
Exon 25	c.5477C>A	p.Ser1826*	Bahubeshi *et al.*[3]

Some of the information is obtained from [https://grenada.lumc.nl/LOVD2/mendelian_genes/home.php?select_db=DICER1]. NM177438.2 was used as a reference sequence.

Our data indicate reduced penetrance of the c.3647C>A *DICER1* mutation. At the time of our study, two of the adult family members showed no signs of neoplasms, whereas the mutation was associated with characteristic tumors at an early age in the proband and her brother. The relatively low penetrance in the described family is in line with other reported familial *DICER1* mutations [3,4,14]. The low penetrance of *DICER1* mutations could relate to a decisive role of miRNAs in the differentiation of stem cells [15], assuming a significant intrauterine selection pressure against the mutations in combination with variations in genetic background. Although the level of penetrance of *DICER1* mutations seems modest, mutational screening of *DICER1* in children and young persons with MNG as well as rare tumors of the ovary, eyes, lungs or kidneys could still be useful. Such screening might also be important in cases devoid of a family history. However, the apparent modest level of penetrance makes the issue of intrauterine testing for the mutation ethically debatable. Genetic counseling of parents of children with a recognized *DICER1* mutation who intend to have more children would benefit from a more precise knowledge of the tumorigenic penetrance of the different *DICER1* mutations. Therefore, clinical follow-up studies of patients with pathogenic *DICER1* mutations with and without neoplastic diseases are required for improved counseling and treatment.

## Conclusions

Very few clinicians couple the rare SLCT with the more common MNG and it is therefore reasonable to assume that the syndromes associated with *DICER1* mutations are under diagnosed. Based on the findings in the present case report, future awareness of SLCT and MNG should reveal a more accurate incidence. Moreover, clinicians should be aware of a potential germ-line *DICER1* mutation when evaluating multinodular goiter in young patients with or without a family history of thyroid diseases.

## Consent

Written informed consent was obtained from the first patient's (Case 1) legal guardian and all other involved patients for publication of this case report and accompanying images. Copies of the written consents are available for review by the Editor-in-Chief of this journal.

## Abbreviations

mRNAs: Messenger RNAs; miRNAs: microRNAs; MNG: Multinodular goiter; SLCT: Sertoli-Leydig cell tumor; US: Ultrasound.

## Competing interests

The authors declare that they have no competing interests.

## Authors’ contributions

MR, TvOH and FCN were involved in the genetic screening of the patients. AJ, CR, MRu and AMG performed the clinical examinations and the genetic counseling of the patients. MR and TvOH drafted the manuscript, while FCN, AJ, CR, MRu and AMG were involved in the revision of the manuscript. All authors read and approved the final manuscript.
